# Forgotten, but not forgiven: facing immunization challenges in the 21 ^st^ century

**DOI:** 10.25100/cm.v49i3.4154

**Published:** 2018-09-30

**Authors:** Ricardo Palacios

**Affiliations:** 1 Division of Clinical Trials and Pharmacovigilance, Instituto Butantan, São Paulo, SP, Brazil.; 2 School of Philosophy, Literature and Human Sciences, Universidade de São Paulo, São Paulo, SP, Brazil.

The creator of several vaccines given to children around the world everyday, Maurice Hilleman, advised that at the same time that new vaccines would emerge in the 21^st^ century due to technological advances, unfounded criticisms of vaccines would extended beyond spurious belief systems to actual anti-vaccine movements. He pointed out that these movements are aimed at disruption of vaccine programmes through use of public media including the press, television and the Internet in his response to the spurious association between autism and Crohn's disease with one of his main creations, MMR vaccine ^1^. Andrew Wakefield, author of an unsound scientific paper in 1998 proposing such association, was motivated by an undue agreement to support a lawsuit ^2^. Nevertheless, Wakefield remains as an outstanding voice in the anti-vaccine movement ^(^ ^3^. 

Why the once feared diseases disappeared from collective memory? On the other hand, adverse events following immunization that we used to bear as a fair risk for the expected benefit are not accepted anymore? Why we have forgotten the benefits, but not forgiven the risks? How could we define new strategies to face the challenges of immunization programmes?

The 1976 swine flu immunization programme in United States was a landmark on the questioning of risk-benefit ratio for vaccines. The concern on a new pandemic flu after triggered a large mass vaccination campaign. Pandemic flu cases did not appear, but serious adverse events did raise questions on public opinion ^4^. This contrasted with most of the vaccines where decreasing incidence of a preventable diseases compared with safety concerns seems to be acceptable for the society. On the other hand, efficacious vaccines, like whole-cell pertussis vaccine (wP), have been also on the spot due to relevant adverse events after immunization. Several scientists in different countries publicly challenged the risk-benefit ratio of wP vaccine with a subsequent drop of immunization coverage leading to re-emergence of pertussis cases. The English case become paradigmatic because vaccine uptake fell from 81% to 31%, but after pertussis cases increased, the coverage raised up to 93% and pertussis cases fell again ^5^. This correlation between vaccine acceptability, disease incidence and adverse events led to propose potential stages in the evolution of an immunization programme ^6^. This framework is one of the basis of the WHO vaccine safety training for immunization programmes ^7^) and it is summarized in Figure 1a.

**Figure 1:**
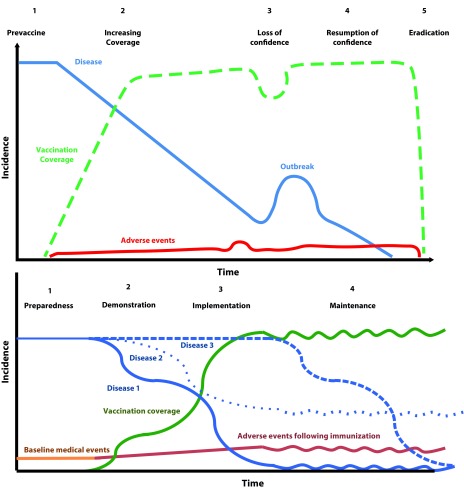
Potential stages in the evolution of an immunization programme. a) Classic model proposed by Chen RT et al (Reprinted from Chen RT, Rastogi SC, Mullen JR, Hayes SW, Cochi SL, Donlon JA, et al. The vaccine adverse event reporting system (VAERS). Vaccine. 1994; 12(6): 542-50. Copyright 1994, with permission from Elsevier ^(^ ^6^ b) Proposed model for a proactive immunization programme

The expected increase of cases following vaccination coverage decrease occurred in several opportunities and allows a very simple and straightforward message to the population: keep vaccinating to avoid disease comeback. Although useful, such model has also potential drawbacks: it relies on the fear of incident cases to regain trust and the threat of increased disease might occur too late. 

Incidence rates in infectious diseases are dependent on the reproductive number, meaning the number of secondary cases infected from a primary case. If this reproductive number is high and latency period is short, the threat of new cases is delivered and the vaccine uptake will be regained at the cost of individuals acquiring the infection and presenting the disease. Nonetheless, it is worth noting that reproductive numbers vary a lot, even for the same disease. For example, measles reproductive number could range between 3.7 and 203.3 according to birth rate, population density and country development status, among other factors ^8^. Unexpected circumstances affect those factors, visiting a crowded Californian theme park is equivalent to a temporary high population density and can result in a larger outbreak ^9^, but measles outbreaks were mainly attributed to vaccine refusal in several reports ^10^. One year after the cluster in California, a State law barred nonmedical exemption of immunization for children in schools, nurseries and daycare facilities. Nevertheless, how many cases are necessary to revert a tendency of vaccine refusal? Can we prevent epidemics/endemics instead reacting to it?

Most of classical vaccines of the expanded programmes of immunization would match with the stages proposed by Chen *et al* ^6^. Nonetheless, other vaccine preventable infectious diseases have a different natural history, either because the reproductive number is low, or because the proportion of asymptomatic individuals is larger than those who are sick, or because the latency period to have an apparent disease is longer. In example, HPV is an infection with lower reproductive number with large infected asymptomatic populations that would result in HPV-related cancers in a limited number of patients several years later. In other cases, vaccine effects are more difficult to measure such us influenza vaccine where is not easy to distinguish influenza-like illness and strain mismatching from vaccine failure affecting public perception of vaccine effectiveness. Then, the model proposed in Figure 1a has limitations for vaccines against these kind of infections. 

## Building another model to understand a 21st century immunization programme

National immunization programmes emerge as a synthesis of different sets of concepts (Table 1) that make them different from other individual healthcare interventions. The assessment to move towards an individual decision might appear incomplete for programmatic purposes. Public health consideration should support whether it is appropriate to extend immunization to a population. Differences in the approaches can even affect immunization schedules ^11^. 

**Table 1:** Main aspects to be considered for individual and public health decisions to immunize

Aspect	Decision to immunize an individual	Decision to extend immunization to a population
*Expected outcome*	Efficacy	Effectiveness
*Primary value*	Autonomy	Social justice
*Social outlook*	Individualism	Collectivism
*Relation to others*	Independence	Interdependence
*Compliance*	Voluntary	Enacted
*Benefits considered*	Direct effects of a vaccine	Indirect and direct effects of a vaccine
*Risks considered*	Individual risk	Individual and bystander risk
*Social body allowing the decision*	National regulatory agency	National Immunization Technical Advisory Groups
*Liability*	Manufacturer	Government compensation programme

Social changes in different countries ^12^ enhance the value of the set of concepts that support individual vaccination in relation to those supporting population-based immunization. This way, the legitimacy of individual decision appears to overcome the public-health decisions. In most cases, individual and public-health decisions still coincide, then immunization programme can obtain high coverage. But in growing amount of cases, programmatic decisions are challenged by individuals.

Currently, internet provides access to such amount of information to an average individual that can match the amount available to healthcare workers ^13^. Some patients pursue to create a symmetrical relationship with healthcare worker despite huge gaps in knowledge and training to critically assess available information. Patients with questions prefer to look for answers available in seconds instead of spending time looking for professional advice. As a result, patients are in front of tons of information with precarious basis for a proper interpretation ^14^. That is the breach where misguided hoaxes find a room. In what have been called “fake news”, the delusional promise of access to an exclusive source willing to disclose what government and media are hiding is the often bait to fish those who are frustrated with the system or have not fulfilled their expectations. Promotion of resilience mechanisms against rumors is not a new task for immunization programmes, but the internet brought an overload of misleading information. 

The current model of potential stages in the evolution of an immunization programme is mainly a reactive proposal. However, several immunization programme officers are already working in a different way ^7^ ^,^ ^15^. Here, a reorientation to a proactive model is proposed in accordance with the Ottawa Charter for Health Promotion: “The responsibility for health promotion in health services is shared among individuals, community groups, health professionals, health service institutions and governments” ^16^. “Shared responsibility and partnership” is stated as one of the guiding principle of the WHO Global Vaccine Action Plan 2011-2020 and determines one of its strategic objectives: “Individuals and communities understand the value of vaccines and demand immunization both as a right and a responsibility” ^7^. Therefore, the proposed stages aim this process of sharing responsibility and should be suitable for vaccines with different effectiveness and against different diseases. 

## Stages in the evolution of a proactive immunization programme

In a proactive immunization model, the proposed stages in the evolution of an immunization programme would be preparedness, demonstration, implementation, and maintenance (Figure 1b). Each one of the stages would have different objectives and stakeholders involved. Most immunization programmes already perform activities described and the purpose here is to organize them into a conceptual model. 

**1 - Preparedness:** This stage can start during the clinical development plan of the product. Health officers can discuss with manufacturers about needs and ideal characteristics of new vaccines. This interaction can result in modifications of the Target Product Profile and design of clinical trials to provide better fit to public health needs. National Immunization Technical Advisory Groups can review periodically available evidence on the diseases and potential vaccines. Mathematical models for introduction scenarios and pharmacoeconomic assessments will support decisions and determine potential target groups for routine and catch-up immunizations, as well as to prioritize clinical development funding. Baseline surveillance of medical conditions associated to potential adverse events is an opportunity to set a reference to assess impact of vaccination on the incidence of such medical conditions. In parallel, key opinion leaders in academic environment and key community leaders are informed on the perspectives of new vaccines to create awareness on the disease to be controlled.

**2 - Demonstration:** Once a new vaccine is approved by the corresponding National Regulatory Agency, a demonstration trial or a pilot study can be a starting point to test vaccine introduction in the field. Manufacturers, academic sector and non-governmental organizations can collaborate with health officers in the setup of this demonstration trial or pilot study as well as in the impact assessment. Involvement of local communities and strategic communication plans are key to build engagement and avoid rumors. The responsibility sharing is also tested at his stage, and social research would inform key indicators at this point.

The expected timeframe for incidence decrease might be short for diseases with high reproductive number, brief latency period, and a highly efficacious vaccine; i.e. measles (Disease 1 in Figure 1b). Vaccines for other diseases might have lower effectiveness, therefore incidence decrease is not easily perceived by population; i.e. influenza (Disease 2 in Figure 1b). In other cases, the disease has a long latency period and /or a low reproductive number and vaccine effect on disease incidence can take a long time to be detected; i.e. HPV and Hepatitis B (Disease 3 in Figure 1b). Other diseases might have a combination of the characteristics above mentioned. Surveillance system should adapt to these timeframes. Realistic expectations in changes in diseases incidence are part of the message for the community

**3- Implementation:** This is the scale-up stage of the new vaccine. At this point, all learnings during demonstration are applied and monitored. Social and qualitative indicators can complement indicators of vaccine coverage and detect any misleading information. Results from the previous stage are also useful as example for other communities. Peer-experience exchange is also a possibility to build trust among healthcare workers and local communities. This stage is when the process of sharing responsibility occurs and advocacy is encouraged. Identification of vaccine resistant groups is desirable at this stage in order to determine assertive resolutions.

**4-Maintenance:** The mid-term and long-term sustainability of the programme depends on how all the stakeholders handle their responsibilities, either as recipients or providers of the immunization ^7^. To reinforce the value immunization in community life through educational institutions, workplaces, religious organizations can continuously boost the message in populations. Transparency and trustable channels to resolve doubts and to take care of those with adverse events are necessary for the population as well as healthcare workers. This is a key part to build resilience to hoaxes and trigger rapid response whenever necessary. Programmes hardly can sustain a continuous high coverage in the long-term. Oscillation can occur as response to increases in adverse events or misinformation. Staff in charge of monitoring the programmes should include social researchers and communication experts that can also support a rapid and proper answer whenever a concern emerges in a community. 

## Conclusions

The classic model of stages in the evolution of an immunization programme has been very useful to explain the introduction of most vaccines into National Immunization Programmes. Nevertheless, this model is reactive and has limitations to explain the introduction of many vaccines. The reactive position proposed in this model was consistent with an approach where patients are expected to comply with the recommendations received from a healthcare worker. Individualism and increased access to information (i.e. the internet) led patients to question this model. Sharing responsibilities with individuals and communities can offer an opportunity for immunization programmes to switch to a more proactive model. Support from other areas like communication and social sciences would be critical to build new immunization programmes to face the challenges in the 21^st^ century.
