# Supplementary material for: Forgotten, but not forgiven: facing immunization challenges in the 21 st century
Source: Colomb Med (Cali). 2018 Sep 30;49(3):189–92. doi: 10.25100/cm.v49i3.4154 (PMC6220486; doi:10.25100/cm.v49i3.4154)
Supplement: Related texts

# Editorial: Olvidada, pero no perdonada: enfrentando los desafíos de la vacunación en el siglo XXI

El creador de varias vacunas, Maurice Hilleman, previo que al mismo tiempo que surgirían nuevas vacunas en el siglo XXI, debido a los avances tecnológicos, las críticas infundadas a las vacunas se extenderían de sistemas de creencias erradas hasta verdaderos movimientos contra las vacunas. Señaló que estos movimientos están dirigidos a la interrupción de los programas de vacunación mediante el uso de medios de comunicación como la prensa, la televisión e Internet en su respuesta a la asociación espuria entre el autismo y la enfermedad de Crohn con una de sus principales creaciones, la vacuna MMR ^1^. Andrew Wakefield, el autor del artículo científico erróneo en 1998 que proponía dicha asociación, fue motivado por un acuerdo indebido para respaldar una demanda ^2^. Sin embargo, Wakefield sigue siendo una voz sobresaliente en el movimiento contra vacunas ^3^.

¿Por qué las enfermedades antes temidas ahora desaparecieron de la memoria colectiva? Por otro lado, ¿ya no son aceptables los eventos adversos posteriores a la inmunización que solíamos asumir como un riesgo justo para el beneficio esperado? ¿Por qué hemos olvidado los beneficios; pero, no perdonamos los riesgos? ¿Cómo podríamos definir nuevas estrategias para enfrentar los desafíos de los programas de inmunización?

El programa de inmunización contra la gripe porcina de 1976 en los Estados Unidos fue un hito en el tema de la relación riesgo-beneficio para las vacunas. La preocupación por una nueva pandemia de gripe provocó una gran campaña de vacunación masiva. Los casos de gripe pandémica no aparecieron, pero los eventos adversos graves plantearon preguntas en la opinión pública ^4^. Esto contrastó con la mayoría de las vacunas en las que la incidencia decreciente de enfermedades prevenibles en comparación con las preocupaciones de seguridad parece ser aceptable para la sociedad. Por otro lado, las vacunas eficaces, como la vacuna contra la tosferina de células completas (wP), también han estado en el foco de discusiones debido a eventos adversos relevantes después de la inmunización. Varios científicos en diferentes países desafiaron públicamente la relación riesgo-beneficio de la vacuna wP con una consecuente caída de la cobertura de inmunización, lo que llevó a la reaparición de casos de tosferina. La situación en el Reino Unido se volvió paradigmática debido a que el uso de vacunas disminuyó del 81% al 31%; pero, después de que aumentaron los casos de tosferina, la cobertura aumentó hasta el 93% y los casos de tosferina volvieron a caer ^5^. Esta correlación entre la aceptabilidad de la vacuna, la incidencia de la enfermedad y los eventos adversos llevó a proponer etapas potenciales en la evolución de un programa de inmunización ^6^. Este marco es una de las bases de la capacitación de seguridad de la vacuna de la OMS para programas de inmunización ^7^ y se resume en la Figura 1a.

**Figura 1:** Posibles etapas en la evolución de un programa de inmunización. a) Modelo clásico propuesto por Chen RT *et al* (Reimpreso de Chen RT, Rastogi SC, Mullen JR, Hayes SW, Cochi SL, Donlon JA, *et al*. The vaccine adverse event reporting system (VAERS). Vaccine. 1994; 12(6): 542-50. Derechos de autor: 1994, con permiso de Elsevier) ^(^^6^ b) Modelo proactivo para un programa proactivo de inmunización

El aumento esperado de casos después de que la cobertura de vacunación disminuye en varias oportunidades trasmite un mensaje muy simple y directo a la población: siga vacunándose para evitar el regreso de la enfermedad. Aunque es útil, este modelo también tiene inconvenientes potenciales: se basa en el temor de los casos incidentes para recuperar la confianza y la amenaza de un aumento de la enfermedad puede ocurrir demasiado tarde.

Las tasas de incidencia en enfermedades infecciosas dependen del número reproductivo, es decir, el número de casos secundarios infectados de un caso primario. Si este número reproductivo es alto y la latencia es corta, la amenaza de nuevos casos se cumple y la cobertura vacunal se recuperará a costa de que individuos adquieran la infección y presenten la enfermedad. No obstante, vale la pena señalar que los números reproductivos varían mucho, incluso para la misma enfermedad. Por ejemplo, el número reproductivo de sarampión podría oscilar entre 3.7 y 203.3 según la tasa de natalidad, la densidad de población y el estado de desarrollo del país, entre otros factores ^8^. Las circunstancias inesperadas afectan esos factores, como visitar un parque temático de California es equivalente a un aumento temporal de la densidad de la población y puede resultar en un gran brote ^9^, pero los brotes de sarampión se atribuyeron principalmente al rechazo de la vacuna en varios informes ^10^. Un año después del brote en California, una ley estatal prohibió la exención no médica de inmunización para niños en escuelas, y guarderías. Sin embargo, ¿cuántos casos son necesarios para revertir la tendencia del rechazo de la vacuna? ¿Podemos evitar que las epidemias / endemias en lugar a reaccionar ante ellas?

La mayoría de las vacunas clásicas de los programas ampliados de inmunización coincidirían con las etapas propuestas por Chen *et al*^6^. No obstante, otras enfermedades infecciosas prevenibles por vacunación tienen una historia natural diferente, ya sea porque el número reproductivo es bajo o porque la proporción de individuos asintomáticos es mayor que la de los enfermos, o porque el período de latencia para tener una enfermedad aparente es mayor. Por ejemplo, el virus del papiloma humano (VPH) es una infección con un número reproductivo bajo con grandes poblaciones asintomáticas infectadas que darían lugar a cánceres relacionados con el VPH en un número limitado de pacientes varios años después. En otros casos, los efectos de la vacuna son más difíciles de medir, como la vacuna contra la influenza, donde no es fácil distinguir enfermedades similares a la influenza y la falla por el desfase de cepas de la vacuna que afecta la percepción pública sobre la efectividad de la vacuna. Luego, el modelo propuesto en la Figura 1a tiene limitaciones para las vacunas contra este tipo de infecciones.

## Construyendo otro modelo para entender un programa de inmunización del siglo XXI.

Los programas nacionales de inmunización emergen como una síntesis de diferentes conjuntos de conceptos (Tabla 1) que los hacen diferentes de otras intervenciones de atención médica individuales. La evaluación para avanzar hacia una decisión individual puede parecer incompleta para fines programáticos. Las consideraciones de salud pública deben apoyar si es apropiado extender la inmunización a una población. Las diferencias en los enfoques incluso pueden afectar los programas de vacunación ^11^.

**Tabla 1:** Aspectos principales a considerar para las decisiones de salud individual y pública para inmunizar

Aspecto	Decisión de inmunizar a un individuo.	Decisión de extender la inmunización a una población.
*Resultado esperado*	Eficacia	Efectividad
*Valor primario*	Autonomía	Justicia social
*Perspectiva social*	Individualismo	Colectivismo
*Relación con otros*	Independencia	Interdependencia
*Cumplimiento*	Voluntario	Promulgado
*Beneficios estimados*	Efectos directos de la vacuna	Efectos directos y indirectos de la vacuna
*Riesgos estimados*	Riesgo individual.	Riesgo individual y espectador.
*Organismo social que permite la decisión*	Autoridad Reguladora Nacional	Grupos de asesoramiento técnico para la inmunización nacional
Responsabilidad	Fabricante	Programa de compensación del gobierno

Los cambios sociales en diferentes países ^12^ aumentan el valor del conjunto de conceptos que apoyan la vacunación individual en relación con aquellos que apoyan la inmunización basada en la población. De esta manera, la legitimidad de la decisión individual parece superar las decisiones de salud pública. En la mayoría de los casos, las decisiones individuales y de salud pública aún coinciden, luego el programa de inmunización puede obtener una alta cobertura. Pero en una cantidad creciente de casos, las decisiones programáticas son cuestionadas por los individuos.

Actualmente, internet proporciona acceso a tal cantidad de información a una persona promedio que puede igualar la cantidad disponible para los trabajadores de la salud ^13^. Algunos pacientes buscan crear una relación simétrica con el trabajador de la salud a pesar de las enormes brechas en el conocimiento y la capacitación para evaluar críticamente la información disponible. Los pacientes que tienen preguntas prefieren buscar respuestas disponibles en segundos en lugar de dedicar tiempo a buscar asesoramiento profesional. Como resultado, los pacientes están frente a toneladas de información con bases precarias para una interpretación adecuada ^14^. Esa es la brecha donde los engaños mal orientados encuentran una habitación. En lo que se ha denominado "noticias falsas" (“fake news” en inglés), la promesa ilusoria de acceder a una fuente exclusiva que está dispuesta a revelar lo que el gobierno y los medios de comunicación están ocultando es el cebo que suele pescar a aquellos que están frustrados con el sistema o que no han cumplido sus expectativas. La promoción de mecanismos de resiliencia contra los rumores no es una tarea nueva para los programas de inmunización, pero Internet generó una sobrecarga de información engañosa.

El modelo actual de etapas potenciales en la evolución de un programa de inmunización es principalmente una propuesta reactiva. Sin embargo, varios oficiales del programa de inmunización ya están trabajando de manera diferente ^7^^,^^15^. Aquí, se propone una reorientación a un modelo proactivo de acuerdo con la Carta de Ottawa para la Promoción de la Salud: “La responsabilidad de la promoción de la salud en los servicios de salud se comparte entre individuos, grupos comunitarios, profesionales de la salud, instituciones de servicios de salud y gobiernos” ^16^. “Responsabilidad compartida y asociación” se declara como uno de los principios rectores del Plan de Acción Mundial de Vacunas 2011-2020 de la OMS y determina uno de sus objetivos estratégicos: “Los individuos y las comunidades entienden el valor de las vacunas y exigen la inmunización tanto como un derecho como un responsabilidad ”^7^. Por lo tanto, las etapas propuestas apuntan a este proceso de compartir responsabilidades y deben ser adecuadas para vacunas con diferente efectividad y contra diferentes enfermedades.

## Etapas en la evolución de un programa de inmunización proactiva.

En un modelo de inmunización proactiva, las etapas propuestas en la evolución de un programa de inmunización serían la preparación, demostración, implementación y mantenimiento (Figura 1b). Cada una de las etapas tendría diferentes objetivos y partes interesadas involucradas. La mayoría de los programas de inmunización ya realizan las actividades descritas y el propósito aquí es organizarlos en un modelo conceptual.

**1 - Preparación:** esta etapa puede comenzar durante el plan de desarrollo clínico del producto. Los oficiales de salud pueden conversar con los fabricantes sobre las necesidades y las características ideales de las nuevas vacunas. Esta interacción puede resultar en modificaciones del Perfil de producto objetivo y diseño de ensayos clínicos para adaptarse mejor a las necesidades de salud pública. Los grupos asesores técnicos nacionales de inmunización pueden revisar la evidencia disponible periódicamente sobre las enfermedades y las posibles vacunas. Los modelos matemáticos para los escenarios de introducción y las evaluaciones farmacoeconómicas respaldarán las decisiones y determinarán los posibles grupos objetivo para las inmunizaciones de rutina y de actualización, así como para priorizar la financiación del desarrollo clínico. La vigilancia basal de condiciones médicas asociadas a eventos adversos potenciales es una oportunidad para establecer una referencia para evaluar el impacto de la vacunación en la incidencia de esas condiciones médicas. Paralelamente, los líderes de opinión clave en el entorno académico y los líderes comunitarios clave están informados sobre las perspectivas de las nuevas vacunas para crear conciencia sobre la enfermedad que debe controlarse.

**2 - Demostración:** una vez que la Autoridad Reguladora Nacional correspondiente aprueba una nueva vacuna, un ensayo de demostración o un estudio piloto puede ser un punto de partida para probar la introducción de la vacuna en el campo. Los fabricantes, el sector académico y las organizaciones no gubernamentales pueden colaborar con los funcionarios de salud en el establecimiento de esta prueba de demostración o estudio piloto, así como en la evaluación de impacto. La participación de las comunidades locales y los planes estratégicos de comunicación son clave para crear un compromiso y evitar rumores. La responsabilidad compartida también se pone a prueba en su etapa, y la investigación social informaría los indicadores clave en este punto.

El período esperado para disminución de la incidencia puede ser corto para enfermedades con un número reproductivo alto, una latencia breve y una vacuna altamente eficaz; por ejemplo sarampión (Enfermedad 1 en la Figura 1b). Vacunas para otras enfermedades pueden tener efectividad menor, por lo tanto la disminución de la incidencia no es fácilmente percibida por la población; por ejemplo influenza (Enfermedad 2 en la Figura 1b). En otros casos, la enfermedad tiene una latencia prolongada y/o un número reproductivo bajo y el efecto de la vacuna en la incidencia de la enfermedad puede tomar mucho tiempo para que sea detectado; por ejemplo HPV y Hepatitis B (Enfermedad 3 en la Figura 1b). Otras enfermedades pueden tener una combinación de las características mencionadas. Los sistemas de vigilancia deben adaptarse a esos tiempos. Las expectativas realistas en los cambios en la incidencia de las enfermedades son parte del mensaje a la comunidad. 

**3 - Implementación:** esta es la etapa de ampliación de escala de la nueva vacuna. En este punto, todos los aprendizajes durante la demostración son aplicados y monitoreados. Los indicadores sociales y cualitativos pueden complementar los indicadores de cobertura de la vacuna y detectar cualquier información engañosa. Los resultados de la etapa anterior también son útiles como ejemplo para otras comunidades. El intercambio de experiencias entre pares también es una posibilidad para generar confianza entre los trabajadores de la salud y las comunidades locales. Esta etapa es cuando se produce el proceso de compartir responsabilidades y se fomenta la promoción. La identificación de grupos resistentes a la vacuna es deseable en esta etapa para determinar resoluciones asertivas.

**4 - Mantenimiento:** la sostenibilidad a mediano y largo plazo del programa depende de cómo todas las partes interesadas manejan sus responsabilidades, ya sea como receptores o proveedores de la inmunización ^7^. Para reforzar el valor de la inmunización en la vida comunitaria a través de instituciones educativas, lugares de trabajo, las organizaciones religiosas pueden impulsar continuamente el mensaje en las poblaciones. La transparencia y los canales confiables para resolver dudas y atender a aquellos con eventos adversos son necesarios tanto para la población como para los trabajadores de la salud. Esta es una parte clave para aumentar la resiliencia a los engaños y desencadenar una respuesta rápida cuando sea necesario. Los programas difícilmente pueden mantener una alta cobertura continua a largo plazo. La oscilación puede ocurrir como respuesta a incrementos en eventos adversos o información errónea. El personal a cargo del monitoreo de los programas debe incluir investigadores sociales y expertos en comunicación que también puedan respaldar una respuesta rápida y adecuada cuando surja una inquietud en una comunidad.

## Conclusiones

El modelo clásico de etapas en la evolución de un programa de inmunización ha sido muy útil para explicar la introducción de la mayoría de las vacunas en los programas nacionales de inmunización. Sin embargo, este modelo es reactivo y tiene limitaciones para explicar la introducción de muchas vacunas. La posición reactiva propuesta en este modelo fue consistente con un enfoque donde se espera que los pacientes cumplan con las recomendaciones recibidas de un trabajador de la salud. El individualismo y el mayor acceso a la información (por ejemplo, vía Internet) llevaron a los pacientes a cuestionar este modelo. Compartir responsabilidades con individuos y comunidades puede ofrecer una oportunidad para que los programas de inmunización cambien a un modelo más proactivo. El apoyo de otras áreas como la comunicación y las ciencias sociales sería fundamental para construir nuevos programas de inmunización para enfrentar los desafíos en el siglo XXI.
